# Annotation of the *M. tuberculosis* Hypothetical Orfeome: Adding Functional Information to More than Half of the Uncharacterized Proteins

**DOI:** 10.1371/journal.pone.0034302

**Published:** 2012-04-02

**Authors:** Tobias Doerks, Vera van Noort, Pablo Minguez, Peer Bork

**Affiliations:** 1 European Molecular Biology Laboratory (EMBL), Heidelberg, Germany; 2 Max-Delbrueck-Centrum, Berlin, Germany; Macquarie University, Australia

## Abstract

The genome of *Mycobacterium tuberculosis* (H37Rv) contains 4,019 protein coding genes, of which more than thousand have been categorized as ‘hypothetical’ implying that for these not even weak functional associations could be identified so far. We here predict reliable functional indications for half of this large hypothetical orfeome: 497 genes can be annotated based on orthology, and another 125 can be linked to interacting proteins via integrated genomic context analysis and literature mining. The assignments include newly identified clusters of interacting proteins, hypothetical genes that are associated to well known pathways and putative disease-relevant targets. All together, we have raised the fraction of the proteome with at least some functional annotation to 88% which should considerably enhance the interpretation of large-scale experiments targeting this medically important organism.

## Introduction

During the last ten years, the cost-efficiency and hence throughput of genome sequencing has increased enormously, and with hundreds of bacterial genomes now available, the quality and comprehensiveness of their annotation is a demanding problem [1]. Although several studies improved functional annotations of large numbers of proteins (e.g. [2], [3]), a considerable fraction of open reading frames is still labeled as “conserved hypothetical protein”, “unknown function” or with similar terms that imply that there is no functional indication for the ORF in question. Improving the functional annotation is of great importance for many follow up studies and we here apply computational tools for function prediction to one of the most devastating human pathogens *Mycobacterium tuberculosis* (MTB), the causative agent of tuberculosis [4]. It is estimated that a third of the world's population has been infected by this pathogen, with approximately one new infection per second [5] resulting in around 9 million new cases of active disease and 1.6 million deaths annually. [6]. Therefore an improved functional annotation of its proteome is of particular urgency.

More than a quarter of the proteome of MTB is categorized as hypothetical in various resources including TubercuList, a database that is regularly updated with new annotations [7]. Many of the “hypothetical proteins” occur in fact in more than one bacterial species, which increases the likelihood that they are indeed protein coding genes and not the consequence of erroneous gene predictions. Proteins that occur in different species can be combined into orthologous groups, which are known to be appropriate for functional analyses and annotations of newly sequenced genomes [8], [9] as orthologous genes tend to have the same functions [10]. In MTB, the bulk of ‘hypothetical’ proteins belong to orthologous groups as defined by eggNOG [11] and is thus amenable to comparative analyses.

For assigning function to hypothetical proteins, homology and orthology-based gene annotation is a well accepted standard [12]. However, novel methods have been developed in the last decade which can complement the classical homology search: these methods rely on functional constraints on genome evolution, and are called ‘genomic context’ approaches; they predict functional associations between protein-coding genes by analyzing gene fusion events, conservation of gene neighborhood, or significant co-occurrence of genes across different species [13]–[21]. Unlike homology-based annotation, which infers molecular features by information transfer from experimentally characterized proteins, genomic context methods predict functional associations *between* proteins, such as physical interactions or co-membership in pathways, regulons, or other cellular processes [22]. Recent large scale analyses automatically assigning GO-terms via genomic context to MTB-proteins provide a first rough insight about function of hypothetical proteins [23]. Here, we combine homology-based function predictions and genomic context analyses (i.e. conserved operon architecture and protein fusion events supported by significant co-occurrence of genes across different species) and evaluate manually the results to drastically reduce the fraction of uncharacterized proteins in MTB.

## Methods

### Set of hypothetical proteins in *Mycobacterium tuberculosis*

From the Tuberculist database [7 (version March 2011)] (http://tuberculist.epfl.ch/) we extracted 1096 genes which were categorized as ‘hypothetical’ or ‘unknown’; for 1079 we identified 913 related ‘clusters of orthologous groups’, COGs, [24], and ‘non-supervised orthologous groups’, NOGs, [25] in the STRING database v8.3 [26].

### Annotation via orthology

The eggNOG database [11] provides an automatically generated functional annotation for each orthologous group, which is based on Gene Ontology, pathway assignment, functional domains and description lines of members of the orthologous groups. If a hypothetical MTB protein was a member of an orthologous group with a functional annotation, this annotation was taken over and manually evaluated. Large scale function predictions derived by homology reduced the number of genes of unknown function to 600, remaining strictly hypothetical (no function annotation or restricted to intrinsic features (eg. alanine rich protein)).

### STRING analysis

Manual in depth genomic context analyses using methods available at the STRING database to detect conserved operon architecture or fusion events supported by co-occurrence of genes across different species were applied to the set of hypothetical MTB- proteins that could not be annotated by orthology. COGs and NOGs, containing the 599 hypothetical MTB-proteins were analysed using the tool STRING (Search Tool for the Retrieval of Interacting Genes/Proteins, http://string.embl.de/) [24], [26], applying a conservative score threshold of 0.4. STRING calculates a ‘confidence score’ on the basis of the three genomic context methods: conserved gene neighborhood, gene fusion events and significant co-occurrence of the genes across a specific subset of species. The prediction accuracy of functional links is often better than the confidence score indicates (for instance when tested against *E. coli* small molecule metabolism [21]. Genomic context networks of the set of hypothetical MTB-proteins were built and each network was inspected manually to prove, whether a hypothetical protein can assigned to a cellular role and to a functional category [7].

### Textmining analysis

We analyzed the entire collection of abstracts in the U.S. National Library of Medicine and the gene and genetic phenotype descriptions from the OMIM (Online Mendelian Inheritance in Man) database. A total of 1,188,757 of these documents were able to be assigned to at least one specific organism and 58,770 of them were associated to *Mycobacterium tuberculosis*. We extracted the gene-MeSH terms co-occurrences from the entire collection of documents analyzed. The co-occurrences are evaluated by means of a confidence score that takes into account the total number of documents in which both elements appear independently and the number of documents in which they are found together to avoid spurious associations. The gene MeSH term co-occurrences were manually evaluated by reading the respective documents.

### Manual analysis

All proteins with an orthology-based predicted functions were manually considered and categorized. Results from text mining and genomic context analysis were manually inspected, conservatively evaluated to predict the most probable functional role and the related category.

## Results and Discussion

### Global statistics

The genome of *Mycobacterium tuberculosis* (H37Rv) contains 4,019 protein coding genes (MTB-genes) [7].

**Figure 1:**
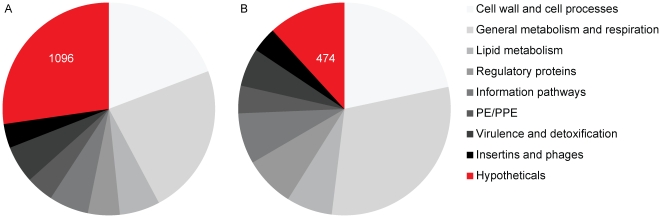
Functional annotation of proteins in the *M. tuberculosis* H37Rv genome. (a) Pie chart: functional distribution before the analysis. (b) Pie chart: estimated functional distribution after re-annotation.

For our analysis, we extracted 1,096 protein sequences from our knowledge best curated database on tuberculosis genomes TubercuList [7], which are categorized as ‘hypothetical’ (note that other databases and depositories containing MTB-genes such as genbank contain more ‘hypothetical’ genes due to missing updates or various other reasons). As this fraction corresponds to more than 25% of the total proteome, we focused on this large dataset to improve functional annotation. In a global search for these 1,096 hypothetical MTB-proteins we retrieved a total of 913 orthologous groups covering 1,079 proteins implying that these proteins occur in at least 2 other species from the STRING database [26]. Several of these orthologous groups, which often contain proteins from many species, contain a few or several functionally annotated proteins in non-MTB-species. Thus, after excluding spurious annotations, we are able to predict functions via orthology transfer for 497 of 1,079 proteins in those groups (Table S1) and categorize them manually (Fig. 1); nevertheless 600 proteins remain hypothetical after functional annotation through orthology.

Of the latter, 582 occur in orthologous groups and are thus amenable to genomic context methods. Using STRING with a medium confidence score threshold of 0.4 used in other studies [2], [21] we build genomic context networks for this set of proteins to visualize operon architectures and fusion events supported by co-occurrence of genes across different species. In depth manual inspection of these genomic context analysis results gives functional information for 122 proteins. Additionally, we apply a text mining procedure [26] to detect additional functions. We analyzed all PubMed abstracts containing “Mycobacterium tuberculosis” or “Mycobacterium” genes for the co-occurrence of MTB-gene names and MeSH terms. All together, the combined analysis of automatically generated functional hints from orthology, genomic context methods and text mining allows us to predict functional hints for 622 of the 1,096 hypothetical MTB proteins (Table S1).

**Figure 2:**
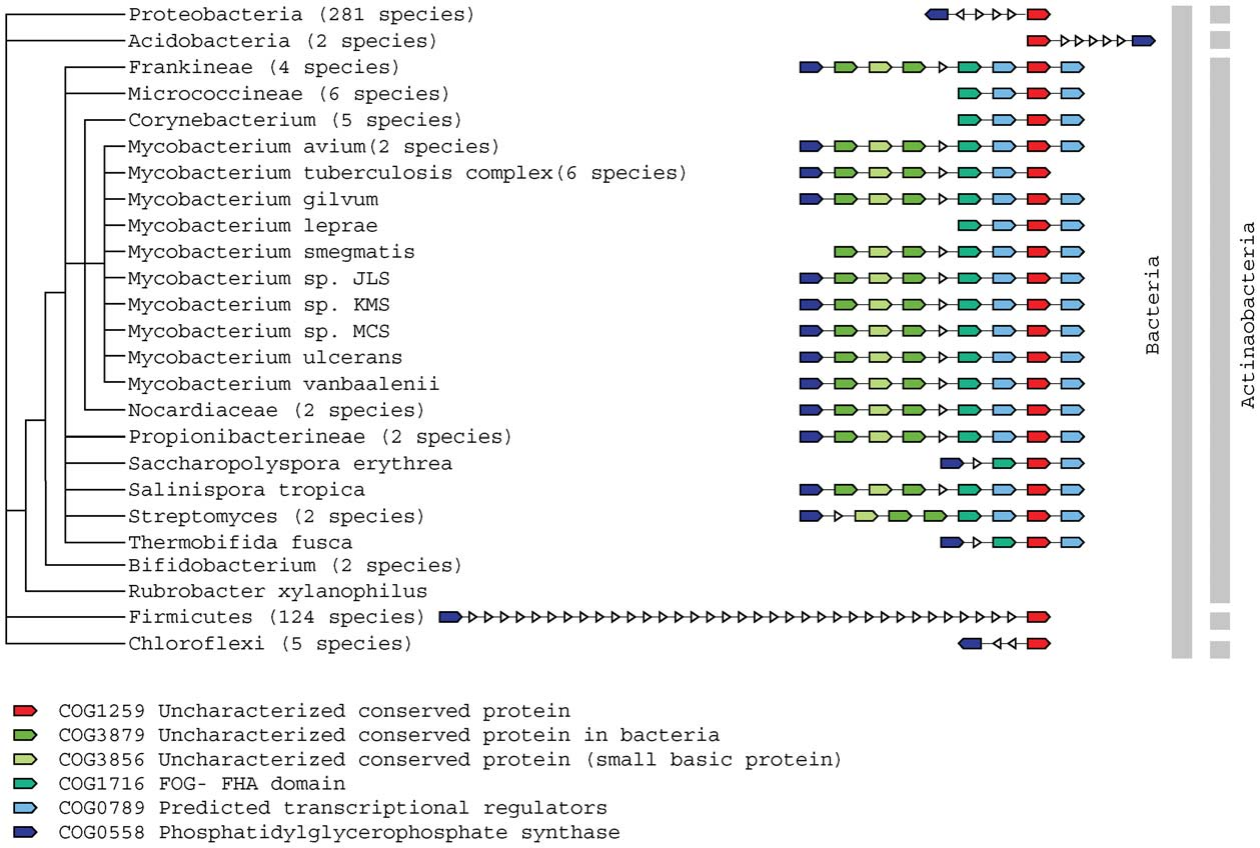
Uncharacterized operon related to cell envelope biogenesis. Species tree with family representatives and corresponding operon architecture. COG1259 (containing the hypothetical gene rv1829 (red unit)) evidentially linked to several uncharacterized genes (variably colored units) to COG0558 (containing PsgA2: Phosphatidylglycerophophate synthase (dark blue unit)).

**Figure 3:**
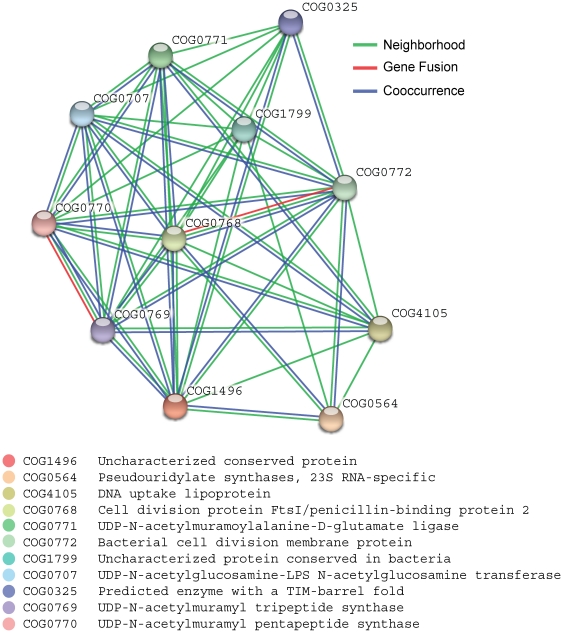
Network of predicted associations for a particular group of proteins, involved in cell wall associated processes (related to COG1496 (containing the hypothetical gene rv2149c (red)). The network edges represent the predicted functional associations. Any edge may be drawn with up to 3 differently coloured lines; these lines represent the existence of the three types of evidence used in predicting the associations. A red line indicates the presence of fusion evidence; a green line - neighborhood evidence; a blue line – cooccurrence evidence.

**Figure 4:**
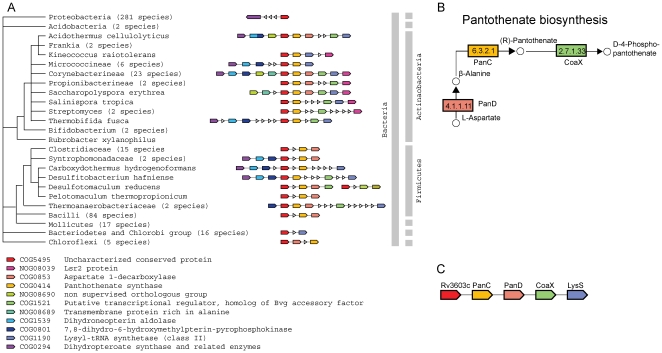
Uncharacterized gene linked to vitamin B5 and B9 biosynthesis pathways. (A) Species tree with family representatives and corresponding operon architecture. COG4595 (containing the hypothetical gene rv3603c (red unit)) evidentially linked to several genes of pantothenate (vitamine B5) biosynthesis pathway (variably colored units upstream the red unit) and to folic acid (vitamine B9) biosynthesis pathway (downstream the red unit). (B) Pathway of pantothenate biosynthesis related to KEGG database [38]. (C) Detailed illustration of the essential genes of the vitamine B5 biosynthesis operon.

The assignments range from new functional modules that comprise several hypothetical proteins associated with functionally annotated proteins, (Fig. 2) to single hypothetical genes in conserved operons encoding well-known proteins (Fig. 3, 4).

The predicted functions belong to a broad variety of cellular activities. These include chromatin-associated processes such as DNA-repair, transcription and translation, metabolic or signaling pathways and membrane associated transport and secretion processes (Table S1).

All together, we provide functional hints for 622 proteins, reducing the set of proteins categorized formerly as hypotheticals/unknown from more than 25% to ∼12%.

Recent studies describe and functionally classify the MTB-proteome using 9 –categories [7], [27]. We classified our functional annotations accordingly to be able to quantify the novelty and its impact on the known functional distribution of the proteome (Fig. 1). Since our analyses reveal function predictions of very different specificity, such a categorization only represents a very rough overview.

As expected, the majority of newly predicted functional proteins is categorized in general metabolism, respiration (∼300 proteins) followed by the less specific category of regulatory proteins (more than 100). Interestingly, the group involved in cell wall associated processes is also mentionable; around 100 functionally predicted proteins are linked to these pathways. 69 proteins are putatively related to chromatin-associated functions/pathways, whereas 34 seems to play a role in lipid metabolism.

Only a very few proteins can be linked to phage origin (7 proteins) or disease relevant detoxification, virulence (6 proteins) and PPE-family (1 protein) (Table S1, Fig. 1).

The following examples illustrate the diversity of the genomic context-based function predictions and highlight a few striking individual predictions.

### A new operon functionally linked to cell envelope biogenesis

The uncharacterized gene *rv1829* (member of COG1259) is located in an operon that is widespread and highly conserved in Actinobacteria (Figure 2). The operon contains several genes with regulatory and membrane-associated functions including *psg*A2, encoding a CDP-diacylglycerol–glycerol-3-phosphate 3-phosphatidyltransferase, which is a member of the Phosphatidylglycerophophate synthase family (COG0558). PsgA2 is a multi-pass membrane protein, which is known to be involved in phospholipid metabolism [28]. It is clearly functionally associated with *rv1829*, the hypothetical cell wall associated gene products of *rv1823* and *rv1825* (see Table S1) and several protein coding genes of less specific functions (Figure 2); the whole operon can be assumed to be functionally important in providing phosphatidylinositol and metabolically derived products. Such products including phosphatidylinositol mannosides, linear and mature branched lipomannan and lipoarabinomannan are reported to play essential roles in the structure and physiology of MTB as well as during host infection [29]. *Rv1829* contains a domain of unknown function called DUF151, which was found to consist of a duplication of two beta(3)-alpha(2) structural repeats, forming a single barrel-like beta-sheet [30], but no function had been annotated so far.

### Assocation of a protein to cell wall synthesis and cell division

An example of a more specific prediction via association to a well-characterized pathway is *rv2149c*, currently annotated as a “conserved hypothetical” protein. *Rv2149c* and its hypothetical orthologs show distant sequence similarity to a broad family of enzymes (multicopper oxidase (laccase) superfamily) [31], which can be involved in different oxidation processes of phenols and diamins and, for example, ensure copper-resistance in *E. coli* [32]. Homology searches indicate that functional residues are conserved. This putative enzyme is a member of COG1496, which is significantly linked to several proteins involved in cell wall formation is present in a variety of bacterial species. In Firmicutes and Actinobacteria, the hypothetical gene is part of an operon which contains cell division genes, for instance *fts*I, *murD*, *fts*W, *mur*G and *sep*F (Figure 3), being essential in cell wall biogenesis and division [28] and known as putative drug targets [33], [34]. Although experimental studies in a single species have shown that orthologs of *rv2149c* are not obligatory essential for viability and growth [35], our analysis reveals a clear involvement of this hypothetical enzyme in cell wall biosynthesis and cell division.

### A putative novel member of the pantothenate biosynthesis pathway

Genes of the pantothenate (vitamin B5) biosynthesis pathway (see Figure 4b) are essential in MTB and are potential antituberculosis drug targets [36].

For instance, pantothenate synthetase (EC 6.3.2.1), encoded by the panC gene (see Figure 4c, COG0414), catalyzes the essential adenosine triphosphate (ATP)-dependent condensation of D-pantoate and beta-alanine to form pantothenate in bacteria, yeast, and plants; pantothenate is a key precursor for the biosynthesis of coenzyme A (CoA) and acyl carrier protein (ACP). Because the enzyme is absent in mammals and both CoA and ACP are essential cofactors for bacterial growth, pantothenate synthetase is an attractive chemotherapeutic target [36]. In our genomic context analysis we detect *rv3603c* (member of COG5495), a gene of unknown function, in a conserved operon with *panC* and other genes of the pantothenate biosynthesis pathway (Figure 4a and b). Interestingly, the hypothetical leucine- and alanine-rich protein Rv3603c appears not only upstream in the vitamine B5 biosynthesis pathway, but is also widespread in Actinobacteria and Firmicutes, where it is significantly associated with another vitamin biosynthesis pathway: the folate biosynthesis (see Figure 4a, eg. COG1539 and COG0294). The genes in those pathways are essential and are highly attractive drug targets [37]. The position of *rv3603c* suggests a regulatory role, putatively forming a link between vitamin B5 and B9 biosynthesis pathways. Therefore, this protein could be an interesting drug target,and is thus an entry point for further experimental exploration.

Taken together, our large scale and manual in depth analysis in *Mycobacterium tuberculosis* provides functional predictions that range from broad biological process assignment to specific molecular functions for 622 of 1096 ‘hypothetical’ genes. Genomic context analyses presumes a classification of a gene into an orthologous group and indeed 1079 of the total 1096 ‘hypothetical’ proteins are assigned to such groups according to the STRING database; for 57% of these, functional hints can be deduced, 45% via homology and ∼12% via genomic context analyses and text mining. The results show the potential of continuous computational function assignment combined with manual inspection and evaluation and considerably extend the functional knowledge on MTB by reducing the fraction of hypothetical proteins by 50%. This will ease the interpretion of systems-wide functional genomics screens and is another step towards the understanding of one of the most baneful pathogens world-wide.

## Supporting Information

Table S1 **Hypothetical MTB-genes and their putative function predicted here.** Column A: gene name; column B: predicted function; column C: functional category; column D: prediction method (orthology, gene fusion, related operon); column E: related orthologous group; column F: prediction source; column G: hyperlink to source page.
(XLSX)
